# Hippocampal MRS and subfield volumetry at 7T detects dysfunction not specific to seizure focus

**DOI:** 10.1038/s41598-017-16046-5

**Published:** 2017-11-23

**Authors:** Natalie L. Voets, Carl J. Hodgetts, Arjune Sen, Jane E. Adcock, Uzay Emir

**Affiliations:** 1 Wellcome Centre for Integrative Neuroimaging, FMRIB, Nuffield Department of Clinical Neurosciences, University of Oxford, John Radcliffe Hospital, Oxford, OX3 9DU UK; 2 Oxford Epilepsy Research Group, Nuffield Department of Clinical Neurosciences, University of Oxford, John Radcliffe Hospital, Oxford, OX3 9DU UK; 3 Cardiff University Brain Research Imaging Centre, School of Psychology, Cardiff University, Cardiff, CF24 4HQ UK

## Abstract

Ultra high-field 7T MRI offers sensitivity to localize hippocampal pathology in temporal lobe epilepsy (TLE), but has rarely been evaluated in patients with normal-appearing clinical MRI. We applied multimodal 7T MRI to assess if focal subfield atrophy and deviations in brain metabolites characterize epileptic hippocampi. Twelve pre-surgical TLE patients (7 MRI-negative) and age-matched healthy volunteers were scanned at 7T. Hippocampal subfields were manually segmented from 600μm isotropic resolution susceptibility-weighted images. Hippocampal metabolite spectra were acquired to determine absolute concentrations of glutamate, glutamine, myo-inositol, NAA, creatine and choline. We performed case-controls analyses, using permutation testing, to identify abnormalities in hippocampal imaging measures in individual patients, for evaluation against clinical evidence of seizure lateralisation and neuropsychological memory test scores. Volume analyses identified hippocampal subfield atrophy in 9/12 patients (75%), commonly affecting CA3. 7/8 patients had altered metabolite concentrations, most showing reduced glutamine levels (62.5%). However, neither volume nor metabolite deviations consistently lateralized the epileptogenic hippocampus. Rather, lower subiculum volumes and glutamine concentrations correlated with impaired verbal memory performance. Hippocampal subfield and metabolic abnormalities detected at 7T appear to reflect pathophysiological processes beyond epileptogenesis. Despite limited diagnostic contributions, these markers show promise to help elucidate mnemonic processing in TLE.

## Introduction

Clinical imaging investigations in drug-resistant temporal lobe epilepsy (TLE) aim to detect seizure foci arising from surgically targetable lesions, the most common of which is hippocampal sclerosis (HS). The presence of HS on diagnostic imaging offers a prognostic marker for surgical treatment success in TLE^1,2^. The complex and heterogeneous architecture of the hippocampus, however, hinders detection of cell loss that may differentially affect specific hippocampal subfields across patients^3^.

High-field 7T MRI provides enhanced contrast relative to standard (1.5 or 3T) clinical MRI systems, alongside exquisite spatial detail due to increased signal-to-noise yield^4^. Consequently, 7T MRI offers potential to identify hippocampal lesions that go undetected in around 20% of routine TLE clinical scans. In people with focal epilepsy, initial 7T studies have demonstrated sensitivity to detect tuberous sclerosis lesions^5^, polymicrogyria^6^, focal cortical dysplasia^7–9^, and hippocampal sclerosis^9–12^. Compared to clinical MRIs, 7T anatomical imaging enables improved qualitative^9,11^ and quantitative^10,12^ hippocampal atrophy ratings for TLE patients with known pathology. But to provide added value, 7T must show enhanced sensitivity to pathology *not* evidenced on clinical MRI that, furthermore, improves treatment outcome predictions *in individual patients*. Yet, in the only prior study of patients with normal clinical imaging, 7T hippocampal volumetry showed no association with post-operative seizure outcomes^12^.

Despite the predictive association between HS and surgical outcomes in TLE, the relationship between seizure activity and neuronal atrophy remains elusive^13^. In 10–30% of surgically resected epileptic hippocampi, no features of sclerosis are identified on pathological examination^3,14^. In those patients whose diagnostic MRI scans appear normal (so-called ‘MRI-negative’ TLE patients), measures of tissue metabolism may instead identify hippocampal dysfunction informative for surgical selection. Non-invasive MRI spectroscopy (MRS), in particular, enables quantification of concentrations for a range of brain metabolites that may inform specific pathophysiological processes in TLE. The majority of MRS studies have focused on N-acetyl-aspartate (NAA), reductions in which have proven informative to detect neuronal dysfunction even in TLE patients with normal-appearing MRI (e.g.^15–18^). Because unbalanced excitatory and inhibitory conductance is implicated in epileptogenesis^19^, quantifying levels of excitatory hippocampal glutamate and inhibitory GABA neurotransmitters has become increasingly important in TLE^20^. Microdialysis studies have uncovered increased glutamate levels from epileptic hippocampi even in the interictal state^21,22^. However, measuring concentrations of glutamate and related metabolites (glutamine, GABA) non-invasively using MRS poses significant technical challenges due to the distribution of their spectral peaks across several resonance frequencies, which, furthermore, overlap with those of other metabolites. Due to the increased signal-to-noise and spectral resolution afforded by ultra-high field imaging, these, among 15 neurochemicals, have now become quantifiable at 7T^23^.

Here, we performed a multimodal imaging study at 7T in 12 pre-surgical TLE patients and age-matched healthy controls. Our aim was to quantify hippocampal neurochemical concentrations, as well as hippocampal subfield volumes from ultra-high (600μm) resolution susceptibility-weighted anatomical scans acquired in pre-surgical TLE patients with either clinically-reported atrophy or normal-appearing MRI. T2* susceptibility effects are pronounced at ultra high field strength, providing novel, exquisite contrast to the internal architecture of the hippocampus^24,25^. We extended conventional group-level comparisons with individual patient-control analyses to evaluate whether neurochemical abnormalities and focal hippocampal subfield atrophy quantified at 7T provide lateralizing data, especially in TLE patients with normal-appearing MRI.

## Results

### Hippocampal sub-region atrophy

**Figure 1:**
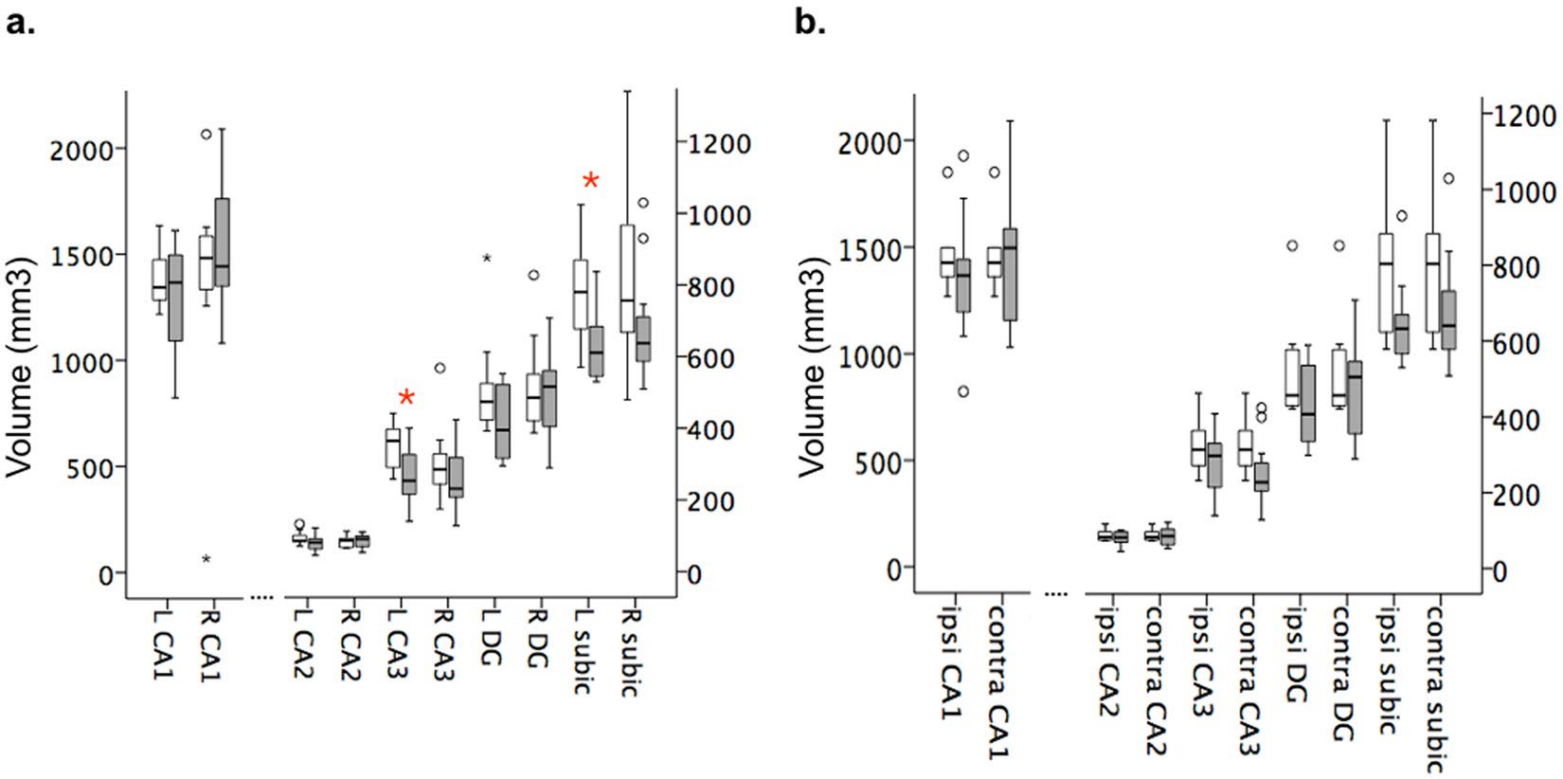
Group-level 7T hippocampal subfield volumes. Hippocampal subfield volumes derived from manual segmentation of high-resolution (0.6 × 0.6 × 0.6 mm) SWI scans acquired at 7T. Individual subfield volumes are presented for groups of controls (white bars) and TLE patients (grey bars). When considered as a group, TLE patients had reduced left CA3 (F = 6.35, p = 0.021) and left subiculum (F = 4.92, p = 0.04) subfield volumes compared to healthy controls (**a**, red asterisks), which did not reach significance when patients were considered according to hemisphere of ictal EEG onset (**b**) (only ipsilateral subiculum retained a tendency to reduced volumes in patients: F = 4.2, p = 0.055). Error bars represent 95% confidence intervals.

Patient clinical and demographic data are presented in Table 1. To evaluate whether hippocampal subfield volumes differed between our TLE patients and age-matched healthy controls, we performed manual segmentations as previously described^26^ of the CA1, CA2, CA3 and dentate gyrus subfields on ultra-high resolution (600μm isotropic) susceptibility weighted imaging (SWI) scans acquired at 7T. Due to artifacts in the SWI scans (see Methods and Supplementary Fig. S1), segmentations could not be performed in one control and were excluded for the left hemisphere of one patient. At the group-level, analyses of covariance, adjusting for age, revealed significantly reduced left hemisphere CA3 (F = 6.35, p = 0.021) and subiculum (F = 4.92, p = 0.040) volumes in TLE patients versus controls (Fig. 1, Supplementary Table S1).

**Table 1:** Patient clinical and demographic data.

Case	Age	Age at onset	F/c	Clinical MRI	Ictal video-EEG	AEDs at time of 7T scan	icEEG findings	PET	Surgical outcome (Engel/ILAE)
**1**	28	9	N	Normal	RTLE	Lamotrigine 200 mg mane, 300 mg nocte	Rhpc	R MTL	1b/2
						Lacosamide 300 mg bd			
						Clobazam 20 g nocte			
**2**	29	7	N	Normal	Bil (L > R)	Levetiracetam 1250 mg bd	Bil	L TL	—
						Lacosamide 100 mg bd			
						Topiramate 300 mg bd			
						Clobazam 10 mg prn for clusters			
**3**	37	5	Y	LHS	LTLE	Levetiracetam 1500 mg bd	—	L TL	—
						Carbamazepine 800 mg mane, 1000 mg nocte			
						Clobazam 10 mg prn for clusters (rare)			
**4**	49	19	Y	RHS	RTLE	Phenytoin 600 mg bd	—	normal	3a, 4
						Clonazepam 2 mg bd			
						Lacosimide 150 mg mane, 200 mg nocte			
**5**	29	21	N	LHS	LTLE	Topiramate 125 mg bd	—	—	1a/1a
						Lamotrigine 100 mg bd			
						Clobazam 10 mg nocte			
**6**	57	30	Y	LHS	LTLE	Pregabalin 125 mg mane, 100 mg nocte	—	—	1a/1a
						Tegretol retard 400 mg mane, 600 mg nocte			
						Topiramate 100 mg bd			
						Clonazepam 1.5 mg nocte			
**7**	42	34	N	LHS	RTLE	Carbamazepine 800 mg bd	—	Subtle R MTL	—
**8**	35	18	N	Normal	RTLE	Levetiracetam 1500 mg bd	—	normal	—
						Oxcarbazepine 300 mg mane, 300 mg midi, 600 mg nocte			
**9**	37	32	N	Normal	LTLE	Levetiracetam 1500 mg bd	neocortical	normal	—
						Lacosamide 200 mg bd			
						Clobazam 20 mg bd			
						Carbamazepine 600 mg bd			
10*	27	23	N	Normal	RTLE	Carbamazepine 400 mg mane, 800 mg nocte	—	Minor R TL	1b/2*
						Perampanel 10 mg nocte			
						Clobazam 20 mg nocte			
**11**	19	16	N	Normal (both small)	RTLE (bilateral interictal)	Levetiracetam 1500 mg bd	—	Bil (R > L)	—
**12**	32	3	Y	LHS	LTLE	Levetiracetam 1500 mg bd	—	LMTL	—
						Lacosamide 200 mg bd			
						Oxcarbazepine 900md bd			

Demographic and clinical data in TLE patients, including age at onset of seizures (in years), presence or absence of a febrile convulsion (F/c), results of intracranial depth electrode (icEEG) investigations, surgical outcome for operated patients and anti-epileptic drug (AED) regime at the time of the scan. All patients were drug-resistant based on lack of response to current and previously tried medications. mg = milligrams. bd = twice daily. Surgical outcomes are reported ≥1 year after surgery, with the exception of Case 10 (asterisk), who was operated 6 months ago. icEEG = intracranial electroencephalography (EEG). HS = hippocampal sclerosis. PET = Positron Emission Tomography. Seizure outcomes are listed according to both the Engel and International League Against Epilepsy (ILAE) classification schemes.

**Figure 2:**
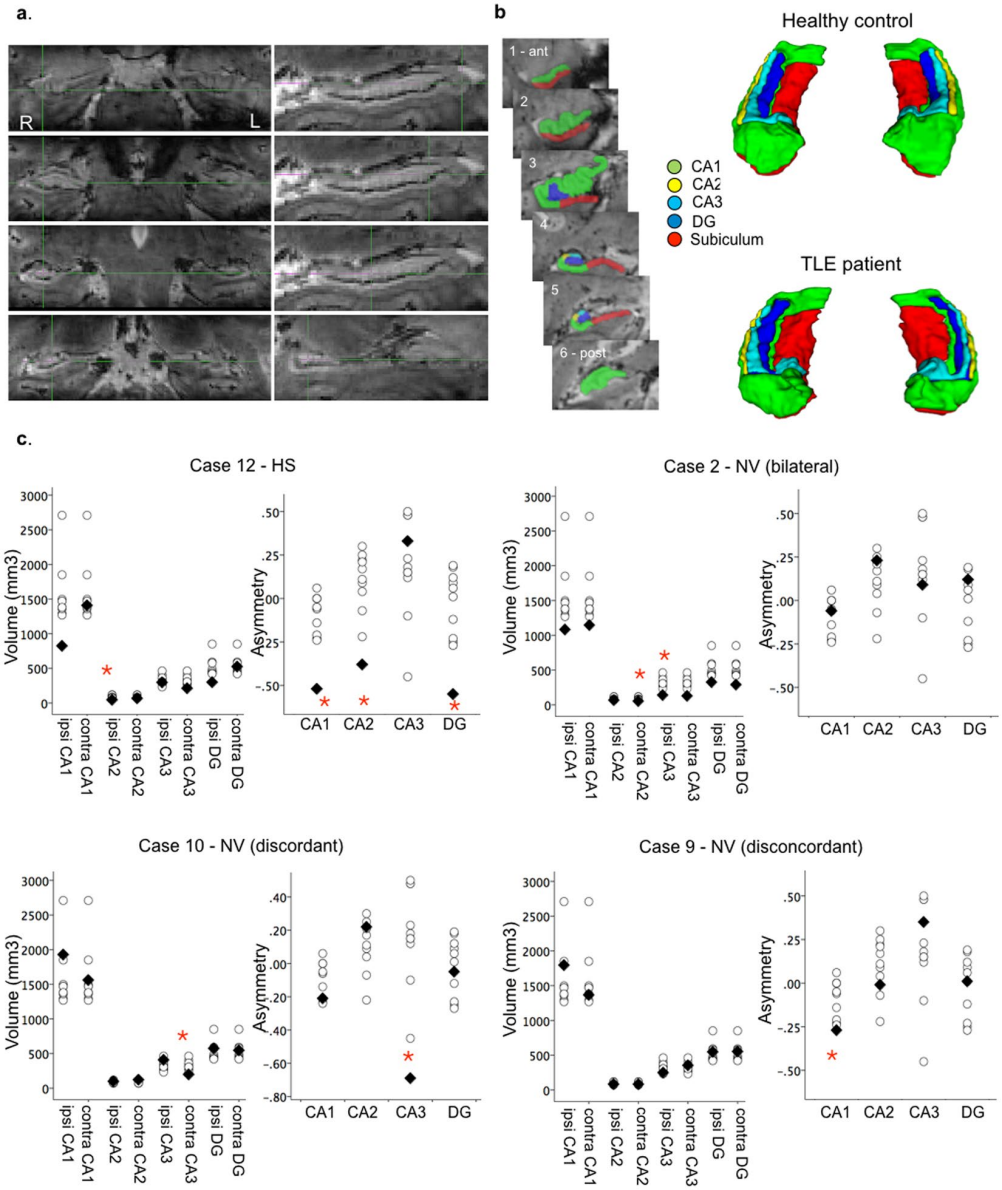
Ultra-high field 7T hippocampal subfield volumetry. (**a**) Ultra-high resolution (0.6 × 0.6 × 0.6 mm) 7T hippocampal imaging in a representative TLE patient. Slices were angled perpendicular to the long axis of the hippocampus for manual subfield segmentations in the coronal plane (**b**). (**c**) Manually defined subfield volumes for CA1, CA2, CA3 and DG as well as calculated asymmetry between the left and right hemisphere, are shown for 4 patients, illustrating the range of individual patient-control volumetric results. Individual patient values (black diamond) are represented overlaid onto the range measured in controls (open circles). Red asterisks mark significant differences in volumes/asymmetries of individual patients when compared to the control group (permutation tests, p < 0.05, uncorrected). Asymmetry in volumes of subfields CA1, CA and DG were identified ipsilateral to the side of seizure onset in a patient with radiologically evident HS (Case 12). Conversely, normal asymmetry but bilateral reductions in subfield volumes were detected in a MRI-negative patient subsequently found to have bilateral seizure onset (Case 2). Discordant findings were, however, identified in Case 10, who showed marked asymmetry driven by a significant reduction in left CA3 subfield volume, contralateral to right-lateralized ictal EEG discharges and right medial temporal PET hypometabolism. This patient underwent a right temporal lobectomy with good outcome at 6 months (Class 1b). Similarly, in Case 9, significant CA1 asymmetry appeared weighted towards the contralateral hemisphere (the raw values of which were not significantly different from controls). Video-EEG in this patient was not localizing within the left temporal lobe and subsequent intracranial recordings indicated a lateral rather than hippocampal seizure onset zone.

To further determine if ultra-high field imaging identified localized subfield atrophy consistent with atypical HS not detected on diagnostic clinical imaging, we evaluated each individual patient’s volumetric measurements against the group of controls through case-control permutation tests using PALM (https://fsl.fmrib.ox.ac.uk/fsl/fslwiki/PALM). In order to increase the number of permutations possible to enable single case-control analyses with small sample sizes, permutations were performed with sign flipping^27^. At the individual level, permutation tests identified altered subfield measures in 9 patients and trends in a further 2. Selected cases are illustrated in Fig. 2. Among the 4 patients with established radiological evidence of HS on clinical MRI, 7T subfield findings were concordant in 3 patients (Cases 7, 12 and 6) and uninformative (i.e. normal) in Case 3. A further 4 patients had equivocal clinical MRI but lateralizing PET abnormalities. In these 4 patients, subfield results identified bilateral pathology consistent with confirmed bilateral seizure onset in Case 2, but were discordant with seizure and PET lateralization in Cases 11, 1, 10. Finally, among the 3 patients with normal diagnostic (MRI + PET) imaging, subfield data were concordant with EEG lateralization in Case 9, discordant in Case 4 and uninformative in Case 8 (Table 2).

**Table 2:** Individual patient clinical imaging data and 7T findings.

TLE	Age	Clinical MRI	vEEG	PET	SWI subfield volumes	SWI asymmetry	MRS concentrations	MRS asymmetry
**1** ^**‡**^	28	Normal	RTLE	R MTL	LCA3, p = 0.03	CA2, p = 0.04 (L < R)	—	—
**2** ^**‡**^	29	Normal	Bil (L > R)	L TL	LCA3 (p < 0.01), RCA2 (p = 0.05)	n.s.	—	—
**3**	37	LHS	LTLE	L TL	n.s.	n.s.	—	—
**4°**	49	normal	RTLE	normal	n.s. (trends for R tNAA p = 0.06; L tNAA p = 0.06)	CA2, p = 0.09 (L < R)	n.s.	Gln p = 0.03 (L < R)
						DG p = 0.04 (L < R)		
**5°**	29	LHS	LTLE	—	n.s. on the R	—	RGlu p = 0.01	n.s.
							RtNAA p = 0.04	
							LGln p = 0.02	
							LGlu p = 0.02	
**6**°	57	LHS	LTLE	—	n.s.	CA2, p < 0.01* (L < R)	—	—
						DG p = 0.01 (L < R)		
**7**	42	LHS	RTLE	Subtle R MTL	LCA3, p = 0.02	n.s.	LGln p = 0.01	n.s.
							LIns p = 0.06 (inc)	
**8**	35	Normal	RTLE	normal	n.s.	n.s.	LGln p = 0.01	n.s.
							LtCho p = 0.03	
							Lins p = 0.02	
							trend LtNAA p = 0.06	
**9** ^**‡**^	37	Normal	LTLE	normal	n.s.	CA1, p = 0.05 (L < R)	LtCho p = 0.01 (inc)	tNAA p = 0.01
							LtCr p = 0.02 (inc)	
							RtNAA p = 0.02	
**10**°	27	Normal	RTLE	Minor R TL	LCA3, p = 0.01	CA3*, p = 0.01 (L < R)	RGln p = 0.02 (inc)	n.s.
							LtCho p = 0.04	
							LtCr p = 0.01	
**11**	19	Normal	RTLE	Bil (R > L)	LCA3, p = 0.02	n.s.	n.s.	n.s.
**12**	32	LHS	LTLE	LMTL	LCA2, p = 0.02	CA1*, p < 0.01 (L < R)	RGln p = 0.044	Glu p = 0.02 (R > L)
						CA2, p = 0.01 (L < R)	RtNAA p = 0.03 Lins p < 0.01 (inc)	Ins p = 0.04 (L > R)
						DG p = 0.012 (L < R)	LtCr p = 0.01 (inc)	tCr p = 0.02 (L > R)

Clinical data in our temporal lobe epilepsy (TLE) patients, presented alongside significant permutation test results (p < 0.05, uncorrected) for manually segmented subfield volumes and metabolite concentrations in the left and right hippocampus in each patient as compared to controls. Values were decreased in patients compared to controls unless marked as increased (“inc”). Asterisks (*) identify results that survived family-wise error correction for up to a maximum of 12 variables. The symbol ^**‡**^denotes patients who underwent intracranial recordings and ° denotes patients who have been operated (see e-Table 1). SWI = susceptibility-weighted imaging. n.s. = not significant. HS = hippocampal sclerosis. vEEG = video electroencephalography. MTL = medial temporal lobe. Glu = glutamate, Gln = glutamine, Ins = myo-inositol, tCho = total Choline (GPC + phosphocholine), tNAA = total N-acetylaspartate (NAA + NAAG).

### Metabolite concentrations

**Figure 3:**
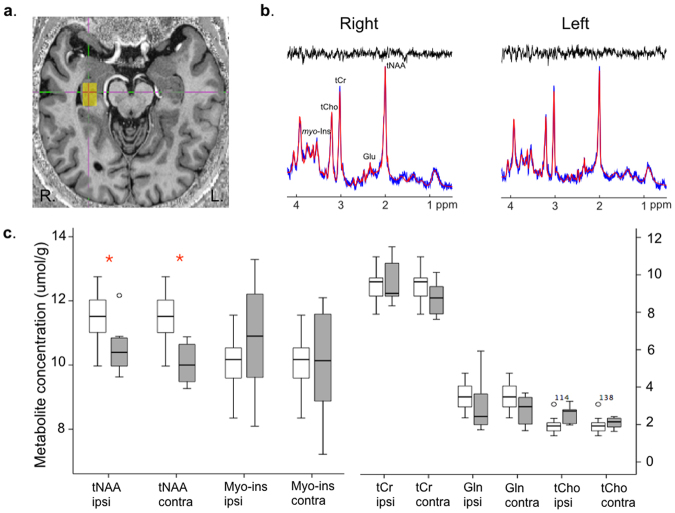
7T hippocampal metabolite concentrations in TLE and controls. (**a**) Voxel placement of the hippocampal region of interest for MRS acquisitions in an example patient, overlaid onto their high-resolution MP2RAGE anatomical image, and representative spectral plots from the left and right hippocampus (**b**). (**c**) At the group-level, TLE patients (grey bars) showed reduced tNAA concentrations compared to healthy controls (white bars) both within the hippocampus ipsilateral and contralateral to the side of ictal onset (red asterisks, analysis of covariance, ipsilateral F = 6.84, p = 0.018; contralateral F = 17.23, p = 0.001). Glutamate is not reported in this hemisphere-clustered analysis due to significant asymmetry (precluding data averaging) in healthy controls. Example single patient results are depicted in Fig. 4. Error bars represent 95% confidence intervals.

We next determined whether metabolite quantification at 7T from left and right hippocampal voxels of interest (VOIs) would identify systematic abnormalities in hippocampal tissue function in TLE patients compared to healthy controls. Concentrations of total NAA, glutamate, glutamine, total choline, total creatine, and *myo*-inositol were measured in 8/11 patients and 12 controls, after data were discarded from 3 patients due to lipid contamination. GABA and lactate concentrations could not be reliably quantified and were excluded from analysis (described in *Methods*, below). As a group, TLE patients showed reduced total NAA levels in both hippocampi (left F = 6.88, p = 0.018; right F = 13.7, p = 0.002, analyses of covariance, correcting for age) relative to controls (Fig. 3, Supplementary Table S2). Other metabolites did not differ between patients and controls when considered at the group level.

**Figure 4:**
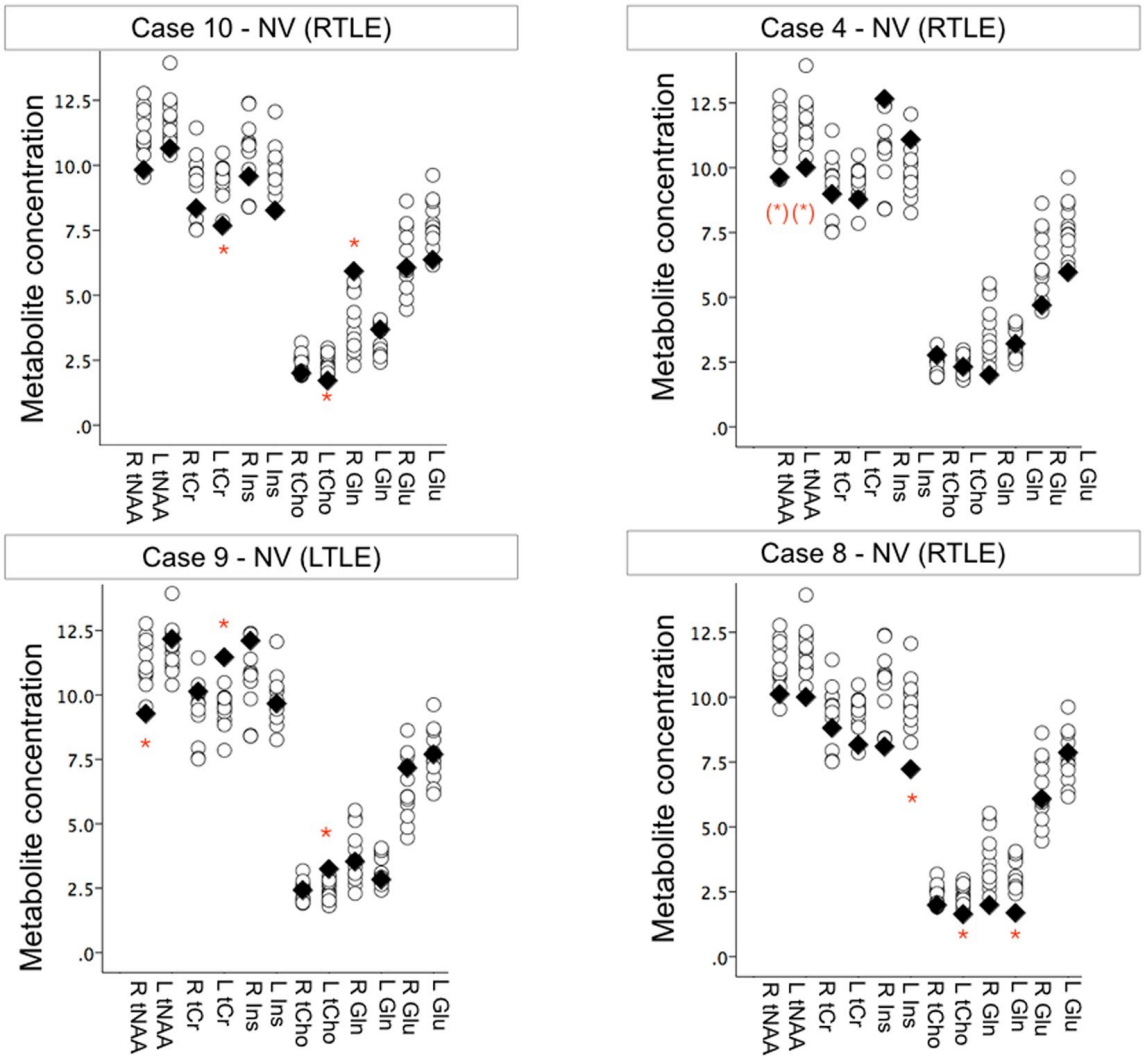
Hippocampal neurochemical abnormalities in individual patients measured with 7T MRS. Absolute metabolite concentrations (μmol/g) estimated using MRS at 7 Tesla from the left and right hippocampus, separately, in 4 TLE patients with normal clinical imaging. Individual patient values (black diamond) are represented overlaid onto the normal range measured in our controls (open circles). Red asterisks mark significant differences in metabolite concentrations measured in individual patients when compared to the control group (permutation tests, p < 0.05, uncorrected for multiple comparisons) (Case 4 showed a non-significant trend. While metabolite concentrations appeared sensitive to detect hippocampal dysfunction not detected on clinical MRI, there was high variability among individual metabolites affected, and no apparent direct link between any single metabolite and lateralization of side of seizure onset.

When compared individually to the group of controls, case-control analyses revealed altered metabolite concentrations in 6 patients and asymmetry in a 7^th^, out of the 8 patients with MRS data. Metabolite differences were concordant with radiological HS in Case 7, but bilateral in Cases 5 and 12. Among the 5 patients who had equivocal clinical MRI, Case 11 had normal metabolite concentrations. The remaining four patients showed neurochemical abnormalities bilaterally (Cases 9 & 10), contralaterally (Case 8) or within the presumed epileptic hippocampus (Case 4). Illustrative individual results are presented in Fig. 4. The individual metabolites affected in each patient are listed in Table 2. In addition to total NAA, altered metabolite concentrations were detected for glutamine (n = 5), total choline (n = 3), total creatine (n = 3), myo-inositol (n = 2), and glutamate (n = 2). No single metabolite provided greater correspondence to clinical imaging and/or EEG than others.

### Associations with epilepsy and memory performance

**Figure 5:**
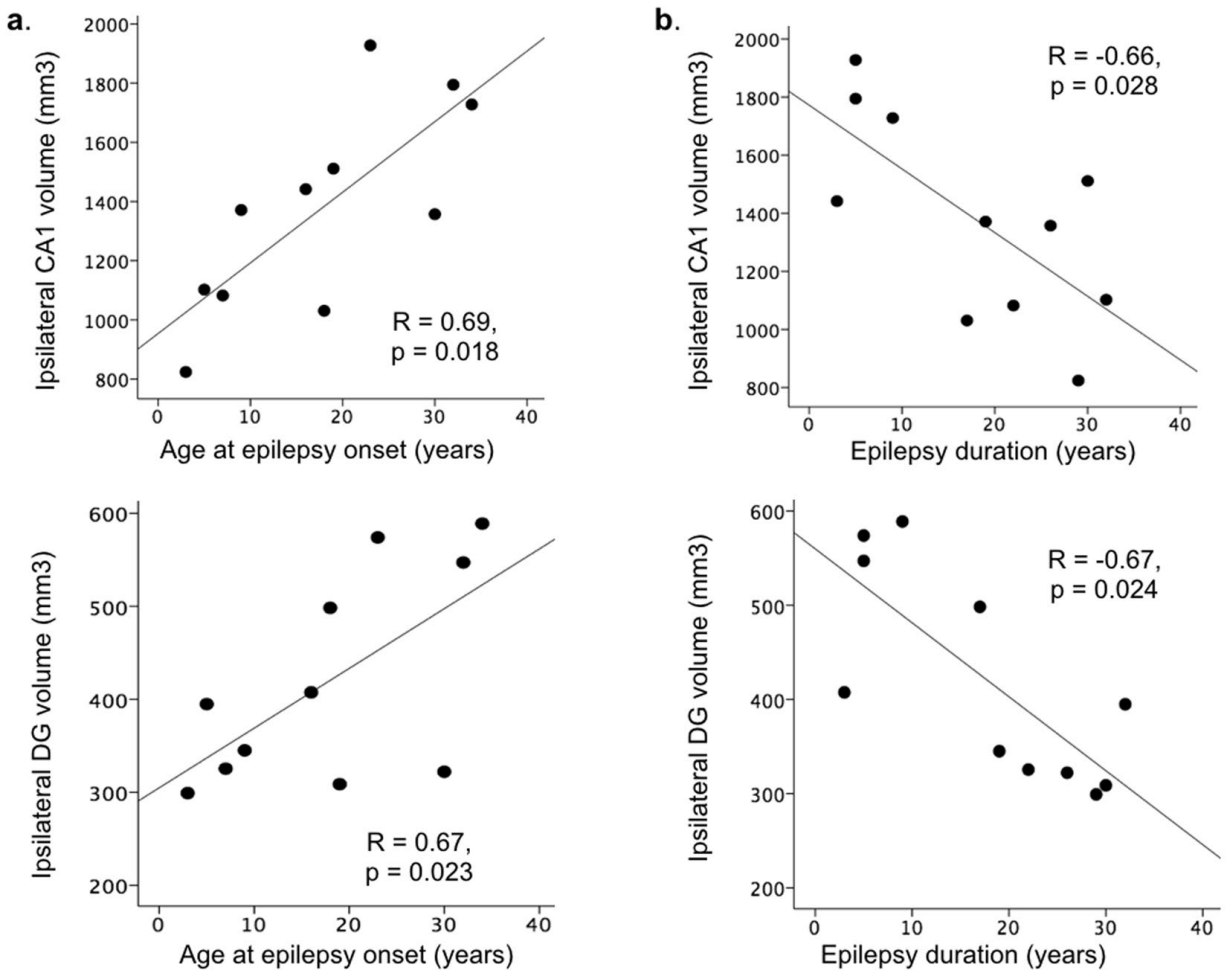
Association between hippocampal subfield volumes and epilepsy variables. Volumes of hippocampal subfields, especially CA1 and the dentate gyrus in the hemisphere of seizure onset showed significant associations with epilepsy-related factors including age at onset of habitual seizures (**a**) and duration of epilepsy (**b**) (Pearson’s two-tailed correlations). As expected, longer disease duration and earlier age at onset of habitual seizures were both associated with smaller hippocampal subfield volumes.

Atrophy of specific hippocampal subfields correlated with clinical disease variables. Younger age at onset of seizures was associated with smaller volumes in ipsilateral CA1 (R = 0.69, p = 0.018) and DG (R = 0.67, p = 0.023) (Fig. 5). Accounting for age, longer epilepsy duration correlated with smaller ipsilateral (CA1, DG, subiculum) and contralateral (CA1, CA2) volumes. Metabolite concentrations showed no such correlations.

**Figure 6:**
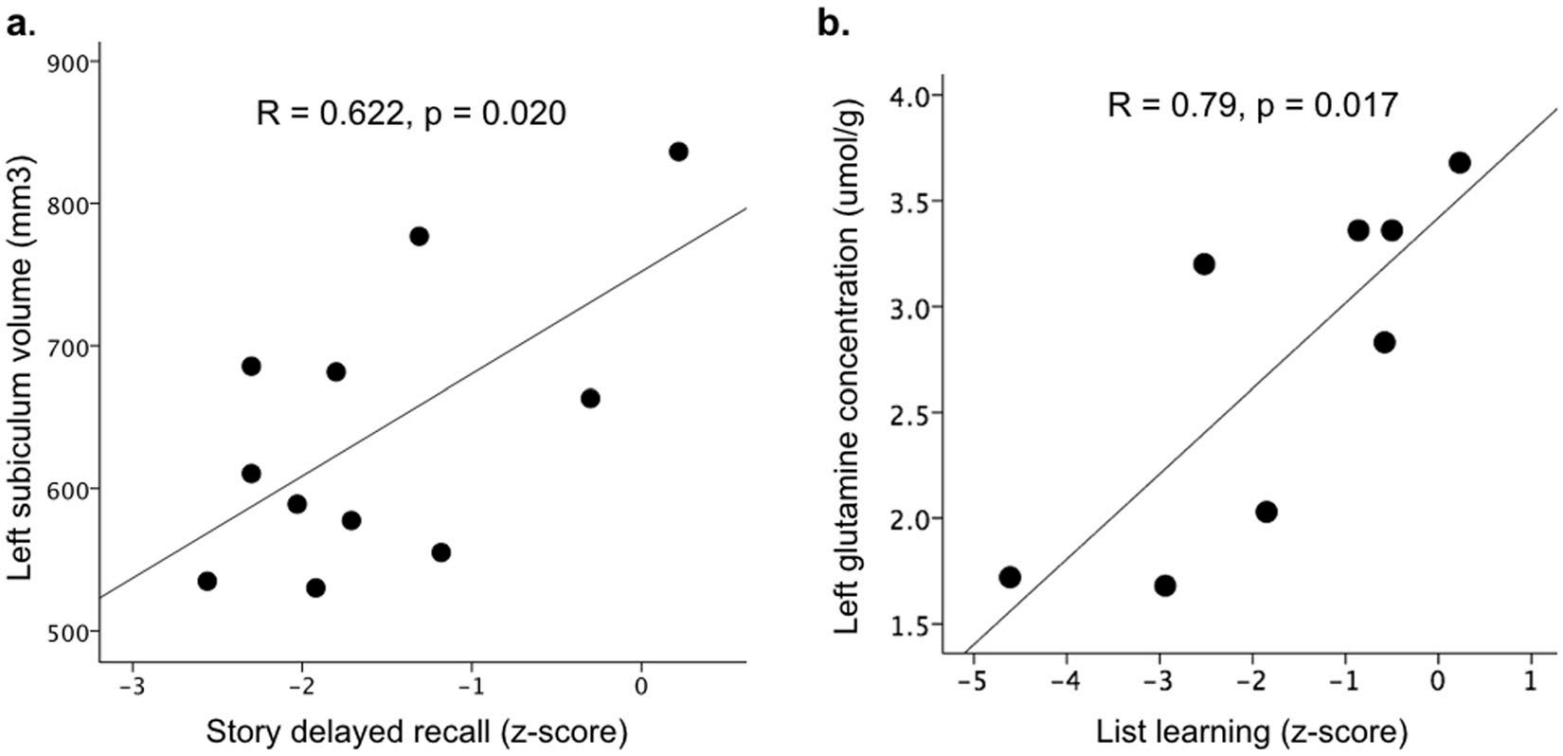
Associations between 7T volumetry, MRS and memory performance. Both volumes of the subiculum hippocampal subfield (**a**) and overall hippocampal metabolite concentrations (**b**) showed significant relationships to clinical neuropsychological test measures of verbal memory performance in TLE patients. Reduced volume of the subiculum in the language-dominant hemisphere correlated with lower delayed story recall (Pearson’s one-tailed correlation), while reduced left hippocampal glutamine concentrations were associated with lower list learning (Pearson’s two-tailed correlation). Significant memory impairment in patients is defined as a test z-score of −1.34 or lower.

Furthermore, in patients, both volumetric and metabolic hippocampal measurements correlated with clinical neuropsychological measures of verbal memory performance. Specifically, smaller subiculum volumes in the language-dominant hemisphere correlated with lower delayed story recall scores (R = 0.62, p = 0.020, one-tailed) (Fig. 6) whereas lower left hippocampal glutamine concentrations correlated with reduced list learning (R = 0.79, p = 0.017, two-tailed). No associations were found between visuospatial memory and right hippocampal imaging measures.

## Discussion

The strong predictive association between HS and surgical seizure control in drug-resistant TLE patients^1,2^ has spurred growing research into the potential of ultra-high resolution MRI to identify pathology not detected at routine clinical field strengths. Accumulating data highlight the increased discernibility at 7T of known epileptogenic lesions^5–11^. The ability of 7T to *newly* identify lesions in patients with ‘normal appearing’ clinical MRI, however, has only rarely been assessed^12^. Our aims were to test whether quantification of subfield volumes and metabolite concentrations at 7T aids in lateralizing hippocampal dysfunction in TLE patients with normal appearing as well as radiologically atrophic hippocampi. Analysis of hippocampal subfield volumes, measured from ultra-high resolution 7T scans, revealed atrophy generally concordant with radiological diagnoses of HS, but provided additional information in only 1 TLE patient (14%) with equivocal clinical imaging. Complementary 7T hippocampal MRS revealed neurochemical disruption in 87% of patients, which was not, however, specific to the side of ictal EEG onset in either radiological HS or MRI-negative patients.

Three prominent patterns of HS are recognized histopathologically. The most common (Type 1) is characterized by extensive cell loss affecting CA1, CA3 and CA4, while atypical variants more selectively affect CA1 (Type 2) or CA4 (Type 3)^3^. Both atypical forms are associated with poorer long-term surgical seizure control than classical HS^13^. Detailed pre-operative anatomical stratification of radiological hippocampal atrophy patterns could, therefore, potentially facilitate surgical decision-making. However, atypical CA1- and CA4-‘predominant’ HS is commonly missed on pre-operative clinical MRI^28^.

Studies conducted to date in TLE patients with a presumed hippocampal focus advocate the sensitivity of ultra-high resolution 7T T2 and T2*-weighted scans to uncover variations in subfield atrophy among patients with established radiological diagnoses of HS^10,11^. In contrast, the only prior *in vivo* 7T study to evaluate subfield volumes in TLE patients with normal-appearing MRI identified abnormalities in only 3/9 MRI-negative patients^12^, and these showed no relationship to surgical outcome. In that study, hippocampal subfield volumes were quantified only from the body of the hippocampus, which may have underestimated localized pathology. Here, we extended subfield quantifications to the full length of the hippocampus and confirmed that 7T subfield volumetry clearly distinguished widespread from focal atrophy. Subfield atrophy ranged from none to widespread in our patients with radiologically indicated HS, while patients with normal-appearing clinical MRI showed subfield loss focused on CA3, and, in one case, CA1. Longer disease duration correlated with greater subfield atrophy especially in bilateral CA1 and ipsilateral DG and subiculum, consistent with prior reports highlighting a link between hippocampal atrophy on MRI and clinical factors including epilepsy duration and, potentially, severity (see^3^). However, patterns of hippocampal subfield atrophy showed limited correspondence to pre-operative seizure lateralization in our series. In 3/4 patients who underwent a unilateral amygdalo-hippocampectomy and remained seizure-free one year following amygdalo-hippocampectomy (Table 1), pre-operative 7T localized atrophy to the contralateral hippocampus. Therefore, while greater subfield volume loss in the context of longer histories of chronic epilepsy support progressive seizure-related degeneration^29–31^, the presence or absence of 7T subfield atrophy alone was in general neither a sufficient, nor a strong, indicator of the laterality of seizure onset.

In contrast, subfield volumetry was potentially informative in two patients. Exclusively contralateral hippocampal atrophy was detected in one patient not rendered seizure-free by surgery, although a sub-complete resection cannot be ruled out. In the second case, bilateral subfield atrophy was detected in a MRI-negative patient with subsequently confirmed bilateral independent seizure onset. 7T imaging has previously shown heightened sensitivity to detect bilateral abnormalities in other epileptic syndromes including polymicrogria^6^. Since the presence of widespread abnormalities reduces the chance of successful surgical seizure control, 7T evidence of contralateral hippocampal damage may help identify patients who are less likely to benefit from surgery. Caution, however, is clearly warranted when interpreting 7T volumetry to inform surgical or intracranial EEG candidate selection, because of the lack of direct link between neuronal loss and seizure lateralization.

TLE patients without any histological features of HS may nonetheless experience significant benefit from surgical intervention. In epilepsy, a leading hypothesis is that the balance between excitatory and inhibitory neurotransmission is unsettled^19^ due to a disruption in the glutamate-glutamine-GABA cycle^21^. Methods able to quantify such metabolic imbalances may, therefore, help localize surgical targets in MRI-negative epilepsies^32^. To our knowledge, a comprehensive quantification of hippocampal metabolite concentrations detectable at 7T, and their complement to subfield volumetry as a means to lateralize TLE has not previously been described.

In our patient group, ultra-high field 7T hippocampal MRS identified bilaterally reduced total NAA at the group-level and decreased glutamine concentrations in 5/8 patients. Reductions in total NAA and NAA/Cr ratios are well established in TLE and have been reported to show sensitivity to hippocampal dysfunction even in patients with normal-appearing MRI (e.g.^15,16,33^). Despite their sensitivity, NAA abnormalities are not always lateralizing (e.g.^34^) and are known to extend contralaterally^15,16,33^. Widespread NAA reductions, furthermore, reportedly revert to normal levels following successful surgical treatment^35,36^, limiting their specificity for seizure localization.

In contrast to well-characterized NAA abnormalities, glutamine and glutamate concentrations have rarely been independently quantified in TLE due to methodological difficulties in resolving these metabolites at clinical field strengths. Using a sequence specifically tailored to detect glutamate, a recent 7T study uncovered asymmetric hippocampal glutamate levels in four TLE patients with normal appearing MRI, driven by heightened signal in the hippocampus associated with seizure onset^20^. We did not find systematic increases in hippocampal glutamate concentrations in our TLE group. A likely source for this disparity is our use of single voxel MRS. Indeed, using a high spatial resolution whole-brain slice approach, Davis and colleagues identified heterogeneity in glutamate concentrations along the anterior-posterior length of the hippocampus, with elevated glutamate signal arising specifically from the head of the hippocampus^20^. Due to the angulation of the hippocampus and to minimize amygdalar tissue inclusion, we positioned MRS voxels predominantly over the body of the hippocampus, which likely diluted sensitivity to heightened anterior hippocampal glutamate levels.

In our TLE patients, a most common, though variable, metabolic imbalance was a reduction in hippocampal glutamine concentrations. While this finding could theoretically provide a marker of deficient glutamine synthesase, recent microdialysis recordings from 41 hippocampal sites in surgical epilepsy candidates unexpectedly revealed no difference in interictal glutamine levels between epileptogenic and non-epileptogenic locations^22^. The possible mechanisms and clinical significance of our observed deviations in interictal hippocampal glutamine concentrations in some TLE patients, therefore, is currently unknown. Due to high estimation errors, we were unable to accurately quantify GABA levels from our hippocampal VOIs at 7T. This technical limitation highlights amplified shimming-related challenges for ultra-high field medial temporal lobe MRS that may be potentially easier to overcome on advanced 3T systems.

The high sensitivity of ultra-high field imaging offers the prospect of generating pathophysiological signatures of individual patients’ unique interictal landscape. Because of substantial variability in deviations of other metabolite levels (namely total choline and myo-inositol) among our patients, we were not able in this study to clarify the mechanistic role of these metabolite abnormalities in chronic TLE. Importantly, however, neurometabolite levels measured from 7T hippocampal MRS in our patients showed no consistent correspondence to side of ictal onset.

The hippocampus is known to play a critical role in aspects of memory formation. The specific computations performed by individual subfields, however, remain the subject of active investigation^37,38^. Previous studies that linked histopathological atrophy patterns to memory skills produced mixed findings. While some reported links between (typically verbal) memory decline and dentate gyrus, but not CA1 cell counts^39^, others found no unique correlations between memory performance and individual subfields^40,41^. We explored whether reduced hippocampal subfield volumes and altered metabolite levels, which were not strongly indicative of side of seizure onset, instead indexed memory function in our TLE patients. Given the heterogeneity in hippocampal subfield atrophy across our HS and normal-appearing patients, the absence of CA subfield and DG associations with verbal memory function in our TLE group is not surprising. Yet, despite our limited sample size, we observed an association between subiculum volume in the language-dominant hemisphere and story recall performance. While subicular neuronal volumes are generally relatively preserved in TLE^42,43^, it has been proposed that subicular cells may be selectively vulnerable to secondarily generalized seizures^44^. Disruption of the subiculum and its major cortical projections, therefore, offers a mechanism for dysfunction of the hippocampal-cortical functional circuitry. Consistent with this idea, it is interesting to note that subiculum neuronal cell counts (indexed by MAP2 labeling) were recently found to be the only predictor of naming declines one year after TLE surgery^41^.

The subiculum has also been proposed as a potential generator of interictal discharges in cell recordings of resected TLE hippocampal tissue^45,46^. Interictal discharge activity directly contributes to memory disruption in TLE^47^ and poses an important confound in evaluations aiming to predict surgical memory outcomes from pre-operative performance. Our data linking subicular volume loss with impaired preoperative story recall may potentially offer an additional means to discriminate transient interictal discharge effects from likely irreversible network disruption in surgical TLE candidates.

Limitations of this study include our small sample size. Sample sizes of between 6 and 13 are characteristic of contemporary 7T epilepsy patient studies, and reflect the cost and challenges of ultra-high field imaging. The stringent contraindication of relatively frequent metallic implants and prior surgical procedures pose a particular recruitment challenge in patients with seizure disorders. We intentionally included TLE patients with complex and ambiguous clinical findings in order to capture those scenarios in which 7T may theoretically be most useful. It was therefore possible from the outset that hippocampal imaging would not detect any abnormalities and yield less sensitivity than prior HS-focused studies. However, among the largest reported series of epilepsy patients to date, visual ratings of extensive 7T imaging also did not provide enhanced discrimination in 19 patients with presumed focal cortical dysplasia or mesial temporal sclerosis and equivocal 3T MRI. Additional limitations include our low number (n = 5) of patients with confirmatory surgical outcomes. Following intracranial investigations, two patients were deemed unsuitable for surgery. One patient declined surgery; one patient declined depth recordings; and two require further investigations to better localize the ictal focus. One additional patient has been operated and is pending clinical follow-up. Finally, owing to selective amygdalo-hippocampectomy being the preferred resection method at our center, comprehensive histopathological analysis of resected tissue was precluded. While daily quality assurance scans were performed to ensure stability in the scanner software and hardware over time, we could not assess scan-rescan reliability from our single measurements. Test-retest data will be important in future studies to ascertain the impact of between-session variability on hippocampal subfield volumetry measurements.

In conclusion, our data indicate high sensitivity but large individual variability in the accuracy with which 7T hippocampal subfield atrophy and metabolic dysfunction lateralize the side of seizure focus in TLE. Neither hippocampal volumetric nor spectroscopic 7T measurements provided a robust predictor of seizure lateralization when assessed against post-operative seizure control. Instead, correlations with memory performance indicate a potential contribution of 7T hippocampal imaging in clarifying the wider impact of chronic epilepsy on elusive memory systems.

## Methods

Twelve pre-surgical patients aged 35.2 ± 10.3 years (range 19–57, 7 men) with suspected medial temporal lobe epilepsy (TLE) were prospectively recruited through the Oxford Epilepsy Surgery Programme (Table 1). The sample size was informed by clinical throughput and those selected for the study were a representative population of patients being considered for selective amygdalo-hippocampectomy. Patients were eligible for the study if they had seizures suspected to arise from mesial temporal structures based on comprehensive multidisciplinary clinical assessment, and were under consideration for epilepsy surgery. A consultant neuroradiologist specialized in epilepsy reported clinical scans, which revealed features consistent with hippocampal sclerosis (HS) in 5 patients and normal or equivocal findings in 7 patients. Three patients required depth electrode recordings (after all other investigations, including research 7T MRI, was complete), due to uncertainty in the hemispheric lateralization or location of seizure onset within the temporal lobe (Table 1). All patients were receiving combination anti-epileptic drug therapy. Controls were 12 healthy volunteers with no neurological or psychiatric history, age-matched to patients (mean 29.5 ± 10.3 years, range: 22–60, 3 men, independent samples t-test p = 0.191). Written informed consent was obtained from all participants in accordance with the principles of the Declaration of Helsinki. The study was approved by the South London Research Ethics Committee.

### Neuropsychology

Each patient underwent comprehensive neuropsychological assessment as part of their surgical evaluation, including test of verbal and non-verbal memory abilities from the Brain Injury Rehabilitation Trust Memory and Information Processing Battery (BMIPB). From this battery, we selected list learning and delayed story recall performance to assess verbal memory and retention, and design learning and figure recall scores to assess non-verbal memory abilities in patients.

### MRI

Data were acquired on a 7 Tesla Siemens MRI system, using a 32-channel head coil at the University of Oxford FMRIB Centre. Barium titrate pads positioned over the temporal bone were used to increase the extent of the effective transmit field (B1+). A whole brain T1-weighted anatomical scan was acquired to inform hippocampal slice angulation and MRS voxel placement.

### Susceptibility-weighted imaging (SWI)

Hippocampal volumetry studies have typically been based on T2 weighted scans that are readily available on clinical MRI systems. With the development of 7 Tesla human MRI scanners, research has focused on utilizing the advantages of higher field strengths to develop optimized and novel signal contrasts that can be exploited for the detection of pathology not easily seen at clinical field strengths. T2* susceptibility effects, in particular, are more pronounced at higher field strengths, providing exquisite contrast to the complex internal structure of the hippocampus^24,25^. Such contrast and high spatial detail can be obtained without the higher power deposition that limits spin-echo based T2 sequences^48^. The origin of the improved contrast remains uncertain, but the larger susceptibility effects have been proposed to reflect variations in iron content between hippocampal subregions^49^, as well as differences in subregion tissue cellularity and microvasculature^25^. Consequently, the majority of previous 7T studies in epilepsy patients have utilized T2*-weighted contrast to investigate hippocampal atrophy. For consistency with prior studies and to harness the advantages of 7T, SWI sequences were collected for manual volumetry in all 12 patients and 11/12 healthy controls using the following parameters: TE = 25.7 ms; TR = 50 ms; voxel size = 0.6 × 0.6 × 0.6 mm; partial Fourier = 6/8; FOV = 192 mm, 44 slices, flow compensation, duration 05:35 minutes. In the 12th control, the SWI scan was acquired at higher resolution, affecting segmentation consistency. This control was therefore omitted from volumetric analyses. Slices were aligned perpendicular to the main axis of the hippocampus for optimal subfield visualization (Fig. 1). SWI scans were acquired twice in all participants, in left-to-right and in right-to-left phase acquisitions. The single best acquisition was selected for manual segmentation in each participant (detailed below).

### Magnetic Resonance Spectroscopy (MRS)

Single-voxel MRS data were acquired in 11 patients and all 12 controls from a 3.36 mL (10 × 12 × 28 mm^3^) voxel region of interest (VOI) placed over each hippocampus. Voxels were carefully positioned to encompass the head and body of the hippocampus while minimizing partial volume contributions from adjacent voxels anteriorly in the amygdala and laterally in the temporal horn and white matter. First and second-order shims were adjusted by gradient-echo shimming, after which first-order shims were fine-tuned using FASTMAP. Spectra were measured with a using a semi-LASER pulse sequence (TR = 6 ms, TE = 36 ms) with VAPOR (variable power and optimized relaxation delays) water and outer volume suppression. In total, 112 averages (duration: 11:12 minutes) were measured separately from the left and right hippocampi.

### Manual segmentation of hippocampal subregions

Hippocampal subfield volumes were calculated from manual segmentations of CA1, CA2, CA3, DG and subiculum (see^26^), blinded to clinical results (Fig. 1). Hippocampal subregions were manually delineated on individual subjects’ ultra-high resolution SWIs using ITK-SNAP (www.itksnap.org). Due to variable degrees of image quality and signal dropout, manual segmentations were performed on the single SWI image that provided the best signal–to–noise in each participant. As described previously^26^, the hippocampus was subdivided into CA1, CA2, CA3, DG and subiculum based on a consensus 7T protocol^50,51^ with reference to previous literature^52–54^. Signal dropout in the medial temporal lobe precluded manual segmentation in one hemisphere of one patient and both hemispheres of one healthy control (Supplementary Fig. S4). These data points were therefore excluded from volumetric analyses. Analyses were performed on the data for the remaining n = 10 controls as well as the right hemisphere data for 12 patients and left hemisphere data for 11 patients.

Hippocampal subfield volumes were corrected for differences in head size using a covariance correction method^55^. First, the slope of the regression line between *total* hippocampal volume and total brain volume (measured using FSL FAST) was calculated in a larger group of 25 previously reported healthy volunteers, scanned on the same 7T scanner using the same SWI sequence^26^. Regression lines were calculated separately for female and male controls, and for the left and right hippocampus. The computed gradient between total hippocampal and total brain volumes from this independent group of controls was then applied to calculate corrected hippocampal volumes for all participant in this study using the equation: Adjusted Volume = Observed Volume − Slope (TBV_subject_ − TBV_mean_), where TBV_subject_ is the total brain volume for the given participant, and TBV_mean_ is the average total brain for the healthy controls. We then, secondly, determined the scaling factor to apply to individual subfield volumes. We divided the observed total hippocampal volume by the adjusted hippocampal volume for each participant, and multiplied each participant’s subfield volumes by this resulting unique scaling factor to obtain head-size adjusted subfield volumes.

To determine the reliability of manual hippocampal subfield segmentations, segmentations in 5 randomly selected healthy controls were performed twice, at least 6 months apart. A repeated-measures ANOVA across all subregion volumes showed no significant effect of time (F = 0.0005, p = 0.98) or interaction between subregion volume estimate and time (F = 0.52, p = 0.72). The intra-class correlation coefficient showed good intra-rater reliability for manually defined volumes, with a mean CI of 0.78 and average 95% confidence interval 0.27–0.97). Only CA2 showed variable segmentation reliability, consistent with prior reports highlighting the greater difficulty in defining the borders of this small subregion (refs^56,51^).

### MRS processing and quantification

Individual spectra were phase and frequency corrected. As previously described^57^, two non-suppressed water spectra were acquired: one for eddy current correction and reconstruction of the phased array spectra (the RF pulses of the VAPOR scheme were turned off) and one for use as a reference for metabolite quantification (VAPOR and OVS schemes turned off in order to eliminate magnetization transfer effects). Single scan spectra from 32 channels were corrected for frequency and phase variations induced by subject motion and then summed.

Metabolite concentrations for total N-acetylaspartate, glutamate, glutamine, total Choline, total creatine, GABA, lactate, and *myo*-inositol were estimated using LCModel^58^, using the unsuppressed water signal as internal reference. To remove the potential impact of cerebrospinal fluid (CSF) on the metabolite concentration estimates, CSF correction was performed on both VOIs. A binary mask of the VOI location was constructed in the same imaging matrix as the T1 structural image and applied to a manual whole-hippocampus segmentation to calculate the voxel grey matter, white matter and CSF fractions within each VOI. Metabolite concentrations were then corrected for the CSF fraction (FCSF) by multiplying the measured values by [1/(1 − FCSF)](LCModel manual).

Metabolites quantified with Cramer-Rao Lower Bounds >50% were considered not reliably detected. On this basis, GABA and lactate were rejected for analysis. For all reported metabolites, CRLBs were ≤25% for every participant, in both hippocampi. Due to lipid contamination, data from 3 patients were excluded from metabolite analysis (leaving n = 8 TLE patients and n = 12 controls).

Signal intensity peaks reflecting metabolite concentrations are typically expressed as ratios normalized to creatine, levels of which are assumed to be homogeneous across brain tissue. However, prior studies in TLE patients identify potential abnormalities in creatine levels within epileptic hippocampi, with reports of increases^59^, reductions^34^ and normal levels^16^ in epilepsy sub-types compared to controls. In our analyses, we therefore, focused on absolute metabolite concentrations.

### Asymmetry calculations

An asymmetry index was calculated for each hippocampal sub-region and each metabolite using the equation (L − R)/[(L + R)/2], where L is the volume or metabolite concentration from the left hippocampus and R represents the equivalent measure quantified from the right hippocampus.

### Group-level statistical analyses

Hippocampal volumetric and metabolite concentration data were compared between groups of patients and controls using univariate analyses of covariance (ANCOVA), controlling for age, in SPSS Statistics (v22). The normality of subfield volume and metabolite data were assessed using Shapiro-Wilk tests for both TLE patients and healthy controls. In controls, subfield volumes fit a normal distribution (all p > 0.05). However, in TLE patients, right hippocampal CA1 as well as left dentate gyrus and CA1 subfield volumes did not meet assumptions of normality. Shapiro-Wilk tests on metabolite concentrations were not significant in TLE patients (i.e. met assumptions of normality), whereas in controls, right hippocampal total choline measurements deviated from a normal distribution. Individual measures that violated the assumptions of normality were subjected to a Box-Cox transformation prior to analysis. Left and right-hemisphere measurements in controls were compared using paired-samples t-tests to determine symmetry prior to averaging for comparison of patients values according to side of seizure presumed onset (ipsilateral/contralateral). Associations between imaging and clinical measures were assessed using two-tailed Pearson’s correlation analyses, except for correlations between hippocampal subfield volumes and verbal memory scores, since hippocampal volumes in TLE patients were always smaller than in healthy controls. Only a positive association between reduced volumes and reduced memory performance were therefore expected.

### Individual-level statistical analyses

Individual-level case-control analyses were performed to assess abnormalities in structural and metabolite measurements from single patients. Because of the possibility that distributions in small control samples might violate the assumptions underlying single-case t-test analyses^60^, we performed inference testing using permutation methods implemented in Permutation Analysis of Linear Models (PALM), part of the FMRIB Software Library package (https://fsl.fmrib.ox.ac.uk/fsl/fslwiki/PALM/)^27^. Variables of interest (the 5 subfield volumes in each hemisphere, the concentrations for our 6 metabolites in each hemisphere, and their corresponding asymmetry indices) were submitted as inputs to PALM, and tested for every patient separately against distributions built from our group of healthy controls. The significance that can be achieved in permutation testing (i.e. 1/n) is fundamentally determined by the sample size. For small-sample and single case-control analyses, PALM offers a solution to this problem in the form of sign flipping. If we can make assumptions that the residuals of the general linear model are symmetrical around zero, then we can randomly ‘flip’ or invert some of the data (making positive values negative), increasing the number of possible data shufflings^27^. The distributions of certain measures such as volume are always positive and skewed. Therefore, we first performed a Box-Cox power transformation on the volume and metabolite measurements to satisfy the assumptions of the model before submitting these to permutation testing. We report permutation p-values for significant results (p < 0.05), uncorrected for multiple comparisons for this first description of combined metabolite and subfield volumetric findings. In results Table 1, findings that survived family-wise error rate (alpha inflation) correction for the number of variables included in each analysis (i.e. up to 5 for subfield asymmetry, up to 6 for metabolite asymmetry, up to 10 for individual subfield volumes and up to 12 for metabolites) are marked with asterisks.

### Data Availability

The datasets generated and analyzed during the current study are available from the corresponding author on reasonable request.

## Electronic supplementary material

Supplementary Information
