# Supplementary material for: Hippocampal MRS and subfield volumetry at 7T detects dysfunction not specific to seizure focus
Source: Sci Rep. 2017 Nov 23;7:16138. doi: 10.1038/s41598-017-16046-5 (PMC5700920; doi:10.1038/s41598-017-16046-5)
Supplement: Supplementary file 1 — Supplementary Information [file 41598_2017_16046_MOESM1_ESM.pdf]

Supplemental Materials for article:

Hippocampal MRS and subfield volumetry at 7T detects dysfunction not specific to seizure focus

Authors: *Natalie L. Voets (PhD) [1, 2], Carl J. Hodgetts (PhD) [1,3], Arjune Sen (PhD) [2], Jane E. Adcock (MD)[1, 2], Uzay Emir (PhD) [1]

Supplemental Table S1. Group-level Hippocampal Subfield Volume Measurements

	Left hippocampus volume (mm3)					Right hippocampus				
	CA1 Mean (s.d.)	CA2 Mean (s.d.)	CA3 Mean (s.d.)	DG Mean (s.d.)	Subic Mean (s.d.)	CA1 Mean (s.d.)	CA2 Mean (s.d.)	CA3 Mean (s.d.)	DG Mean (s.d.)	Subic Mean (s.d.)
**CON (n=10)**	1501.3 (442.4)	93.8 (19.2)	352.0 (59.1)	510.3 (144.0)	782.9 (147.2)	1635.7 (439.6)	83.9 (16.4)	305.2 (107.4)	520.6 (135.3)	832.9 (245.6)
**TLE (n=11/12)**	1281.2 (254.7)	81.7 (24.4)	***268.8** (78.1)	411.4 (107.9)	***635.8** (103.6)	1520.4 (307.6)	84.9 (19.0)	263.5 (91.2)	487.6 (116.4)	682.0 (152.3)

Legend. Mean and standard deviation (s.d.) of volumes for individual hippocampal subfields in healthy controls and patients with temporal lobe epilepsy (TLE), derived from manual segmentation of ultra high resolution (0.6 × 0.6 × 0.6mm) susceptibility weighted MRI scans acquired at 7 Tesla. CA1 - 3 = Cornu Ammonis subregion 1, 2, 3. DG =dentate gyrus. Subic = subiculum. CON = Controls. TLE = Temporal Lobe Epilepsy. Volumes in bold font and marked with asterisks (*) denote volumes that were significantly reduced in TLE patients when compared to healthy controls ($p < 0.05$).

Supplemental Table S2. Group-level 7T Hippocampal Metabolite Concentrations 7T Hippocampal MRS

	Metabolite	Controls (n=12) mean (s.d.)	TLE (n=8) mean (s.d.)
**Left hippocampus**	Total NAA	11.79 (0.93)	***10.73** (0.67)
	Glutamate	7.66 (1.01)	6.63 (0.89)
	Glutamine	3.26 (0.60)	2.73 (0.81)
	Total Creatine	9.45 (0.66)	9.42 (0.54)
	Myo-inositol	9.85 (0.97)	10.30 (1.92)
	Total Choline	2.36 (0.35)	2.36 (0.54)
**Right Hippocampus**	Total NAA	11.27 (0.91)	***9.85** (0.52)
	Glutamate	6.37 (1.26)	6.08 (1.61)
	Glutamine	3.76 (1.09)	3.01 (1.40)
	Total Creatine	9.38 (1.18)	8.92 (0.78)
	Myo-Inositol	10.40 (1.40)	10.62 (1.64)
	Total choline	2.39 (0.38)	2.27 (0.35)

Legend. Group average absolute metabolite concentration estimates (and associated standard deviations, s.d.) from the left and right hippocampus in healthy controls and patients with TLE. Spectra were acquired from two 10 × 12 × 28mm volumes of interest, placed over the left and right hippocampus, respectively. Metabolite concentrations were estimated using LCModel, using an internal water reference. Total NAA = NAA + NAAG; Total Creatine = PCr + Creatine; Total Choline = GPC + Phosphocholine. Metabolites in bold font and marked with asterisks (*) denote metabolite concentrations that were significantly different between TLE patients when compared to healthy controls ($p < 0.05$). Please see also Table 2 in the main manuscript.

Supplemental Figure S4. Artifacts in 7T hippocampal SWI scans excluded from volumetric analyses.

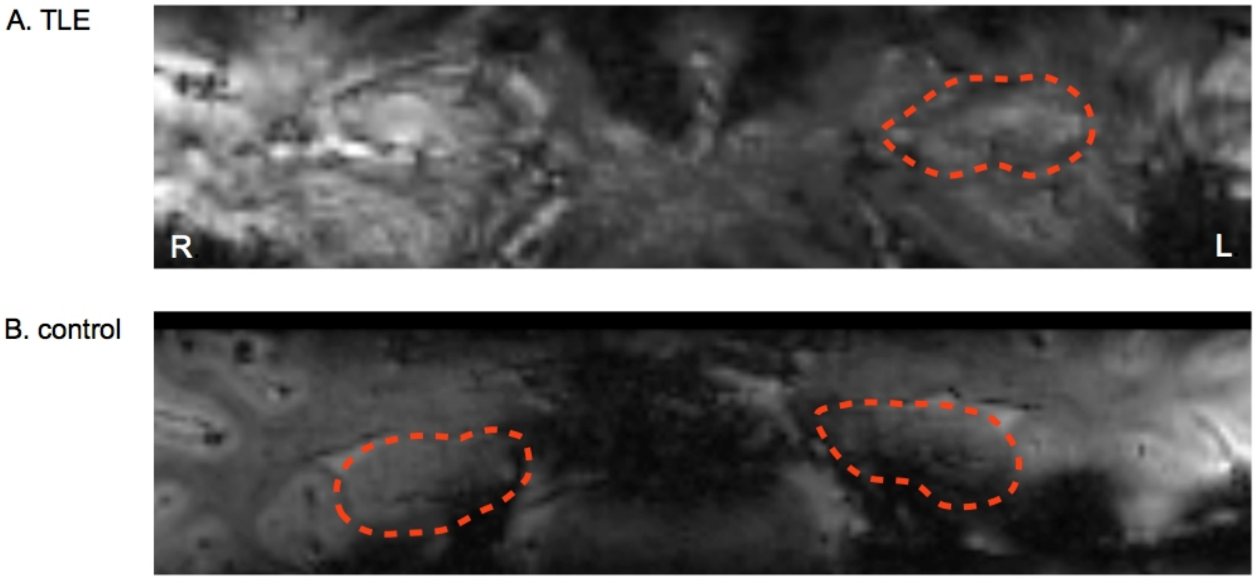

Coronal slice depicting artifacts in SWI scans for a TLE patient (a) and healthy control (b), precluding reliable subfield segmentation. The left hippocampus (red circle) in the epileptic patient is obscured by residual flow artifact. In the control participant, hippocampal segmentations could not be completed due to substantial susceptibility artifact (areas of signal drop-out affecting inferior portions of the hippocampus, circled in red). Please compare against Figure 1, which depicts equivalent slices in a representative successful scan.
